# Changing patterns in deforestation avoidance by different protection types in the Brazilian Amazon

**DOI:** 10.1371/journal.pone.0195900

**Published:** 2018-04-24

**Authors:** Tomas Jusys

**Affiliations:** Euromonitor International, Vilnius, Lithuania; University of Vermont, UNITED STATES

## Abstract

This study quantifies how much deforestation was avoided due to legal protection in Legal Amazon in strictly protected areas, sustainable use areas, and indigenous lands. Only regions that are protected *de jure* (i.e., where deforestation is avoided due to effective laws rather than remoteness) were considered, so that the potential of legal protection could be better assessed. This is a cross-sectional approach, which allows comparisons in terms of avoided deforestation among the different types of protection in the same period. This study covers three different periods. Regions protected *de jure* were sampled by estimating a threshold distance at which deforestation starts to diminish and retaining all pixels up to that distance, and deforestation that has been avoided due to legal protection was estimated by matching. Indigenous lands avoided the highest percentage of deforestation during the 2001–2004 and 2005–2008 periods, followed by those under strict protection and sustainable use areas, in respective order. Shifting patterns in deforestation avoidance are clearly noticeable for the 2009–2014 period when 1) strictly protected areas outperformed indigenous lands in terms of the percentage of saved forests, 2) some protected regions began to attract deforestation instead of avoiding it, and 3) sustainable use areas, on average, did not avoid deforestation.

## Introduction

Forests provide several environmental services, including mitigating greenhouse gas emissions; providing water for human consumption, irrigation, and energy production; conserving biodiversity; and providing scenic beauty for recreation and ecotourism [1]. Deforestation jeopardizes all of these services. The protected area system represents a key measure for protecting valuable ecosystems from deforestation. In Brazil, The National Protected Areas System (SNUC) defines and regulates protected area categories at federal, state, and municipal levels, dividing them into two types: strictly protected, which prohibits resource use and often physical access except for tourism and scientific research; and sustainable use, which permits controlled resource extraction, such as hunting and rubber tapping as well as human settlements [2, 3]. The third type of protected area–indigenous lands–is meant to protect the livelihood of indigenous people; such protected areas are governed by indigenous communities.

The paradigm of sustainable use areas was developed during the 1992 World Parks Congress. Since that time, large territories have been designated as sustainable use areas both in Brazil and around the world. Critics of the sustainable use concept argue that it devalues conservation biology, undermines the creation of more strictly protected reserves, inflates the amount of area in reserves, and places people at the center of the protected area agenda at the expense of biodiversity [4]. Others stress that forest-dependent communities, including indigenous peoples, have stronger incentives than government agencies to protect their livelihood against external deforestation pressures [5, 6].

The objective of this research is to estimate avoided deforestation in strictly protected areas, sustainable use areas, and indigenous lands, but with a novelty: only accessible regions are considered. The goal of the study is to compare deforestation avoidance rates among different governance types in each of the three periods: 2001–2004, 2005–2008, and 2009–2014. Timeframe divisions are based on important events and policy changes that affected deforestation patterns in Brazil. The first division is motivated by the implementation of the 2004 action plan to prevent and control deforestation (PPCDAm, *Plano de Ação para a Prevenção e o Controle do Desmatamento na Amazonia Legal*) which led to a significant decline in deforestation rates. This plan included the creation of five million hectares of forest reserves, from November 2004 to March 2005, in the hotly contested landscape of central Pará [7]. The second division is based on significant constraints that were put on Brazilian farmers which were the result of 1) the global financial crisis, which lowered the demand of agricultural commodities, and 2) voluntary agreements signed by meatpacking companies (Terms of Adjustment of Conduct, TAC, and G4) in 2009 to stop purchasing meat from properties with illegal deforestation.

Empirical evidence on how the three protection types compare in terms of deforestation prevention in Brazil provides no single answer. Earlier studies have suggested that a similar percentage of deforestation or fires is avoided under the three types of legal protection [8, 9], but there is also evidence that strictly protected areas generally avoid more deforestation than sustainable use areas, while indigenous lands avoid the most deforestation in high pressure areas [10]. Also, the performance, in terms of deforestation avoidance rates, of the three types have been found to change over time [11]. Since protected areas are generally located in places that face lower deforestation pressure [12], those studies and other similar investigations [13–20] used matching methods to isolate the effect of protection against deforestation. This was done by comparing protected regions with the most similar unprotected regions based on factors that explain deforestation, such as distances to roads, cities, or land suitability for agriculture.

A sizeable body of deforestation literature finds strong links between agriculture and deforestation [21–24]. Specifically, Brazilian farmers gain land for their cattle and crop cultivation by clearing forests. Thus, the national and international demand for agricultural commodities has a direct effect on deforestation rates in the Brazilian Amazon. To control for the agricultural influence on forest clearings, this study used an index of agricultural suitability and distance to markets (travel time to the nearest city). The road network is another major factor of deforestation. It gives access to migrants and entrepreneurs with different levels of economic resources and plays a decisive role in the dynamics of frontier expansion in the Amazon [25]. Frontier colonization increases the value of the land, stimulating real-estate speculation, and consequently, expansion of deforestation [26]. This study used distances to the nearest official and to the nearest unofficial roads to capture the effect of road infrastructure. Undoubtedly, deforestation patterns are affected by state and national policies, such as rural credit schemes and environmental fines. Small scale analyses cannot capture these effects. Nevertheless, this investigation used the most exhaustive list of potential deforestation drivers available at a fine scale, which have been picked based on previous research [10, 11, 19], academic literature on deforestation drivers [21–25], and data availability. Evidence from empirical studies on deforestation and its drivers [27, 28] also suggest that different factors contribute to deforestation at varying extents in different regions within the same country. Having said this, it is entirely possible that the same type of legal protection may perform differently in different areas of Legal Amazon, even though other factors explaining deforestation are similar.

The magnitude of avoided deforestation, defined as a percentage or area of forest that was not cut because land is legally protected, depends largely on deforestation pressure (the extent to which an area is prone to deforestation). Some protected areas are located near deforestation hotspots (e.g., near large agricultural regions, highly urbanized trade centers, or high-speed roads). Some lie in regions where little deforestation occurs (e.g., in distant rural areas, where forests are cut down only to gain land for subsistence farming), while other protected areas are merely cartographic, i.e., demarcated on maps but almost completely isolated from human access. Avoided deforestation is likely the highest in protected areas that face the highest deforestation pressure and is zero in protected areas that are inaccessible. Therefore, a distinction between *de facto* and *de jure* protection is often made. The former implies that a protected area is protected by its remoteness and inaccessibility; the latter avoids deforestation through effective legislation, i.e., laws that prevent deforestation.

Most earlier research into deforestation avoidance [13–20] has not differentiated between *de jure* and *de facto* protection. This study focused on regions protected *de jure*, which are defined by accessibility via roads and rivers. Rivers were not considered for reasons explained in the Materials and methods section, but in short, rivers open a relatively small area of forests for deforestation. So, focusing on roads, this study filtered accessible areas using a methodology pioneered by Barber and colleagues [29]. Roads in Legal Amazon can be classified into official and unofficial. Official roads are built or funded by national or state governments for geopolitical and economic purposes [30]. These roads often spur a large network of endogenous (unofficial) roads deep into forests. Unofficial roads are built by local interest groups attracted to the Amazon by state policies, market conditions, and other factors, as well as to gain access to land, timber, and other natural resources [31].

The estimates of avoided deforestation in regions protected *de jure* offer a better representation of the potential of legal protection for at least two reasons. Firstly, only territories that can be deforested are included in the analysis. If a large portion of the protected area system is covered by inaccessible forests, avoided deforestation estimates may be low just because most protected lands face zero deforestation *de facto*, but not because legal protection is ineffective. Secondly, strictly protected areas are generally located in regions with lower deforestation pressure [10, 11, 12, 18] or, equivalently: more areas with a strict protection designation are inaccessible. Therefore, avoided deforestation estimates of alternative protection types are inflated relative to those corresponding to strict protection if the distinction between accessible and inaccessible territories is ignored. This may lead to the false conclusion that strict protection areas have less potential to avoid deforestation than sustainable use areas. This is not just a theoretical consideration. A study on the legal protection of forests in Brazil [10] found that the deforestation that was avoided during the 2006–2010 period was the highest for indigenous lands, followed by sustainable use areas and then strictly protected areas in parks created in or prior to 2000. However, strictly protected areas avoided significantly more deforestation than other types of legal protection when considering all parks created in or prior to 2005; the reason for this is that some large strictly protected areas were created in places with high deforestation pressure (large *de jure* and small *de facto* areas), and large territories of sustainable use areas and indigenous lands were created in regions with very low deforestation pressure (small *de jure* and large *de facto* areas). Therefore, filtering accessible territories may alter conclusions, even though it does not nullify the differences between deforestation pressure among different governance types.

## Materials and methods

**Fig 1:**
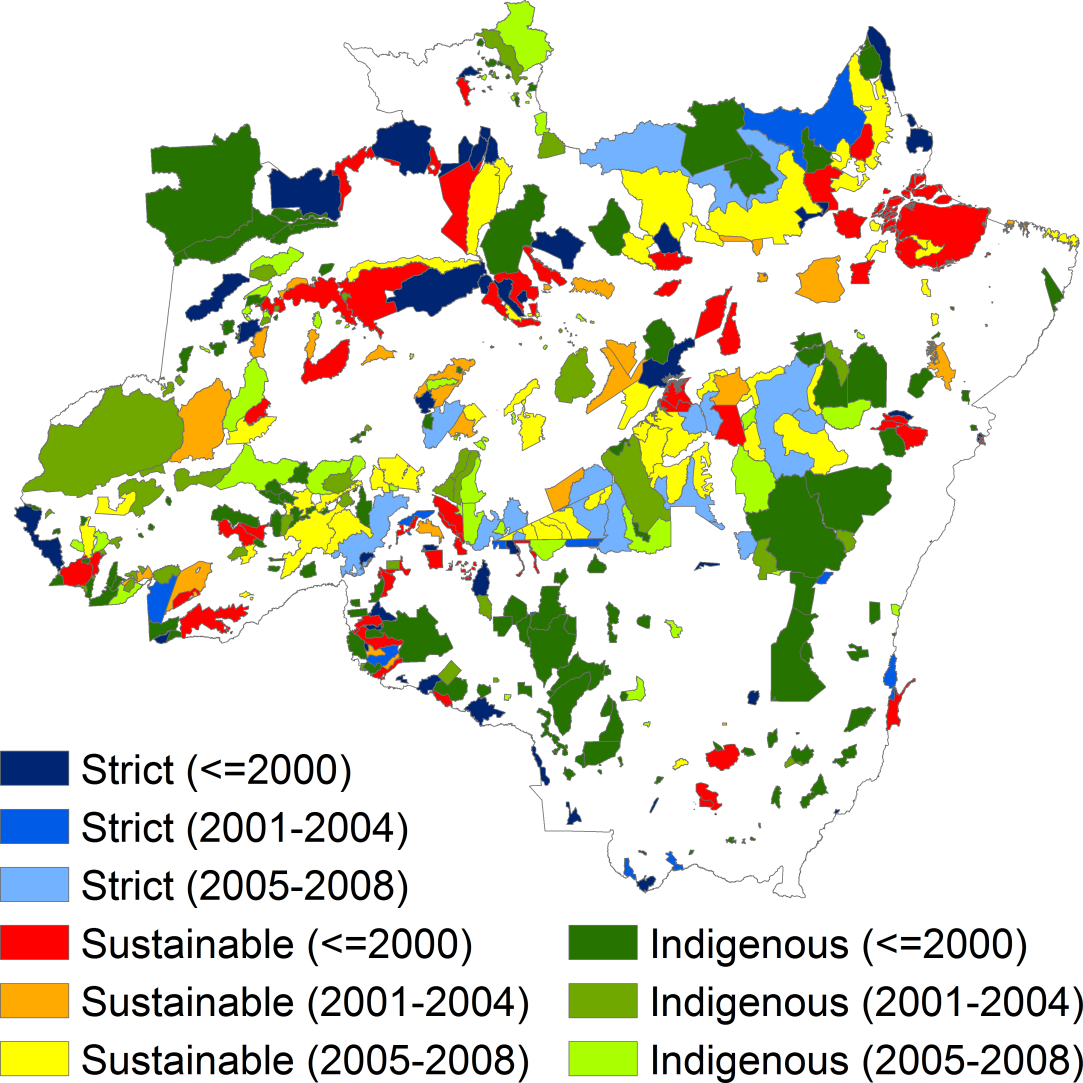
Protected areas in seven states of Legal Amazon by year of creation (in parentheses) and type of governance. Only protected areas larger than 100 km^2^ and created prior to or in 2008 are shown on maps. Military areas are not shown. Projection: Albers equal-area conic.

The study used data on 11 variables, including deforestation (outcome variable), elevation, slope, forest cover, precipitation, agricultural suitability, travel time to the nearest city, the shortest Euclidean distances to the forest edge, official roads, unofficial roads, and rivers (see Table 1 for variable descriptions, units of measurement, and data sources). ArcGIS (Version 10.1, ESRI, Redlands, CA, USA) was used to convert projections onto Albers equal-area conic and to process all data into 1-km spatial resolution rasters, each containing almost 4.5 million cells. Data processing steps are explained and descriptive statistics for parcels are presented in the Supporting Information (S2 File). The shapefiles of protected areas were sourced from Brazil’s Ministry of Environment (see Fig 1 for a geographical visualization of protected areas by type and year of establishment).

**Table 1:** Description of the variables.

Variable	Units	Description	Source
deforestation	%	Percentage of cleared 60-m^2^ parcels in each square kilometer	INPE
elevation	meters	Average elevation over 90-m^2^ parcels in each square kilometer	SRTM-NASA
slope	degrees	Computed from elevation data in ArcGIS	SRTM-NASA
forest cover	%	Percentage of forest cover	NASA
forest edge	meters	Average Euclidean distance to forest edge over 60-m^2^ parcels in each square kilometer	INPE
precipitation	millimeters	Multiyear average of precipitation	World-Clim
agricultural suitability	-	Measure of climate, soil, and terrain constraints for agriculture in 2002	IIASA
official roads	meters	Euclidean distance to the nearest official road built up to 2010	IMAZON
unofficial roads	meters	Euclidean distance to the nearest unofficial road built up to 2010	IMAZON
rivers	meters	Euclidean distance to the nearest river	PNLT-MT
travel time	minutes	Travel time in minutes to the nearest city with more than 50,000 inhabitants in 2000	GEMU-JRC

INPE: Brazil’s National Institute of Space Research (*Instituto Nacional de Pesquisas Espaciais*).

SRTM-NASA: Shuttle Radar Topography Mission-National Aeronautics and Space Administration.

IIASA: International Institute for Applied System Analysis.

IMAZON: Amazon’s Institute of Man and Environment (*Instituto do Homem e Meio Ambiente da Amazônia*).

PNLT-MT: National Plan of Logistics and Transport-Ministry of Transport (*Plano Nacional de Logística e Transportes*-*Ministério dos Transportes*).

GEMU-JRC: Global Environment Monitoring Unit—Joint Research Centre of the European Commission.

This study used a raster of land use classes distributed by Brazil’s National Institute of Space Research (INPE) and a shapefile of official and unofficial roads provided by the Amazon’s Institute of Man and Environment (IMAZON) along with a methodology, proposed by Brandão & Souza [32], to define accessible zones. The INPE’s land use classes include deforestation by year (first in 1997 and then from 2000 annually), forests, hydrography, unforested lands, and clouds. The rasters are created under the PRODES project by digital image processing and visual interpretation of ~30-meter resolution LANDSAT ™ imagery on computer screens, and they are distributed at a ~60-meter spatial resolution. The IMAZON’s vector data consists of official, unofficial, and settlement roads in seven Brazilian states, including Acre, Amazonas, Amapá, Mato Grosso, Pará, Rondônia, and Roraima. The spatial extent of this shapefile also defines the spatial extent of this study. The majority of roads were mapped in 2003, and updates were made up to 2010 using the methodology described by Brandão & Souza [32].

All 60-m^2^ deforestation pixels were allocated either to roads or rivers using the shortest Euclidean distance as the allocation criterium, i.e., if a deforestation pixel is located at a shorter distance to the nearest road as opposed to the nearest river, it was assumed that deforestation had occurred due to road access. Following Barber et al. [29], deforestation pixels allocated to road access were used to estimate the distance at which deforestation penetration started to diminish (4.1 km). This figure was interpreted as a separator between accessible and inaccessible zones, and thus between *de facto* and *de jure* protection (computations are explained in the S2 File). In this way, the accessible zone was defined as an 8.2-kimometer-wide corridor with road segments in the center. This zone did not include lands made accessible by rivers due to difficulties in defining such regions. Occasionally, deforestation associated with rivers penetrated deeply into forests, but in most cases river shores remained undisturbed. Therefore, no single distance that encompasses regions made accessible by rivers could be defined. This approach of separating accessible and inaccessible zones has a limitation: some deforestation happens beyond the distance at which deforestation starts to diminish. Therefore, by conditioning the sampling on that distance, the approach oversamples parcels with a higher propensity for deforestation activity. Thus, avoided deforestation estimates are expected to be somewhat inflated due to this imperfection.

Inaccessible parcels, parcels with sparse forests (<20%), severe cloud contamination (>20%), parcels located near protected areas (<10 km), and those with protected area borders (i.e., not fully protected or unprotected) were discarded. Lands with poor accessibility or small (<100 parcels) protected areas as well as lands with protected areas created during a study period were excluded. Data samples corresponding to the 2001–2004, 2005–2008, and 2009–2014 periods contain 0.99, 0.81, and 0.94 million cells, respectively, out of which, in respective order, 90.5%, 91%, and 81.2% fell in the control sample (refer to S1 File for the data).

The effect of legal protection on deforestation was decoupled from the effects of other contributing factors by propensity score matching using *R*’s library *Matching* [33]. It is a semiparametric approach. To begin with, all different characteristics that affect deforestation must be converted into a single dimensionless score, also known as the propensity or similarity score. For this purpose, this study used a logistic regression with treatment dummy (1 if a land parcel is protected and 0 otherwise) as the dependent variable. Predicted probabilities of this model are propensity scores. Matching itself is non-parametric. For each treatment cell the algorithm finds a certain number of control cells with the most similar characteristics to that treatment cell, i.e., finds control cells with the most similar propensity score. This study selected the three most similar control cells for each treated observation. Matching was done with replacement, meaning that different treatment observations were occasionally paired with the same control observation. Also, ties were broken randomly. A tie occurs when two or more matched control parcels have the same score; a tolerance parameter is used to define what is considered a tie. A caliper of 0.25 standard deviations was imposed to remove poor matches. A caliper is the distance which is acceptable for any match. A tight caliper may lead to much better matches, but it may drop a significant number of treatment observations. Since those observations are not dropped randomly, patterns discovered using a tight caliper may not represent the patterns of the whole sample. Contrarily, not using a caliper may lead to difficulties in filtering the protection effect from the effects of other factors, because poor matches are kept. Once control pairs are identified, avoided deforestation is estimated as the difference between the deforestation rate in a treated cell and the average deforestation rate in matched control cells. Then, all estimates corresponding to land parcels falling in a particular area of interest (e.g., strictly protected lands) are averaged to get the average treatment effect on the treated (ATT).

Before matching, each observation was randomly assigned to one of ten representative subsamples. Matching was done for each group separately, and the results were later averaged. This was done for two reasons. Firstly, logistic regression assumes that observations are independent. This is very unlikely to hold for fine scale spatial data, e.g., precipitation in a land parcel can be explained by precipitation in the neighboring land parcel. Randomly dividing sample into subsamples spaces out the data, i.e., the average distance between land parcels is greater and, therefore, the dependencies between observations are weaker. Secondly, splitting a sample into ten subsamples substantially shortens computational time.

A caveat of the methodology used in this study is that all factors were treated exogenously. Although most factors are indeed exogenous, the link between protection status and roads, and especially logging roads, is clearly endogenous, i.e., the network of logging roads can affect the decision of where protection status is granted and, at the same time, protection status itself may affect the proliferation of logging roads. Since endogeneity is ignored in estimation (no valid instrument can be found at 1-km scale to counteract the problem), model parameters are biased, which in turn means that the algorithm may select control cells with dissimilar characteristics as pairs and miss out on better matches because the score is incorrect. However, if the algorithm succeeds in finding similar control cells despite treating all covariates exogenously, this model imperfection does not have a sizeable adverse effect.

One metric to measure the similarity between the treatment sample and the matched control sample is standardized mean difference (SMD), which in the library *Matching* is calculated as $\left({\bar{x}}_{j}^{T}-{\bar{x}}_{j}^{MC})/sd(x_{j}^{T}\right)$ i.e., as a difference between mean values of covariate *j* in the treatment and the matched control samples, divided by the standard deviation of covariate *j* in the treatment group. SMD equal to zero would imply that the characteristics in the treatment and the matched control samples are identical, i.e., perfect matches. The higher the SMD, the more dissimilar the characteristics are. Although there is no well-established cut-off mark, below which matching is considered successful, the 10% SMD mark is often taken as an indication of marginal difference in the means [34]. However, even if observed characteristics are very well matched, differences in unobserved factors that explain deforestation may bias the estimates. Suppose that matching is conditioned on agricultural factors only, not considering the road layout. Then, if proper matches are found, it only means that the difference in deforestation rates between the treatment sample and the matched control sample is not attributable to agricultural factors, but this difference can be explained both by differences in the road network and other factors, not included in matching, and by legal protection. Therefore, the effect of legal protection cannot be filtered from the effects of the road network and other factors, not included in matching. Deforestation depends on various social, economic, political, and cultural factors. Some of these factors are unobserved and may be very different on protected and unprotected lands. Even though it is not feasible to account for all possible characteristics that affect deforestation, especially in fine scale analyses, this study controlled for ten major factors, thereby significantly reducing the bias.

To sum up the methods, firstly, the methodology developed by Barber and colleagues was applied to define the accessible zone; the remaining parcels were removed. Next, the matching protocol was used to estimate avoided deforestation in different protection types, different periods, and different regions. Finally, standardized mean differences were analyzed to assess the quality of matching.

## Results

### Accessible zone

**Fig 2:**
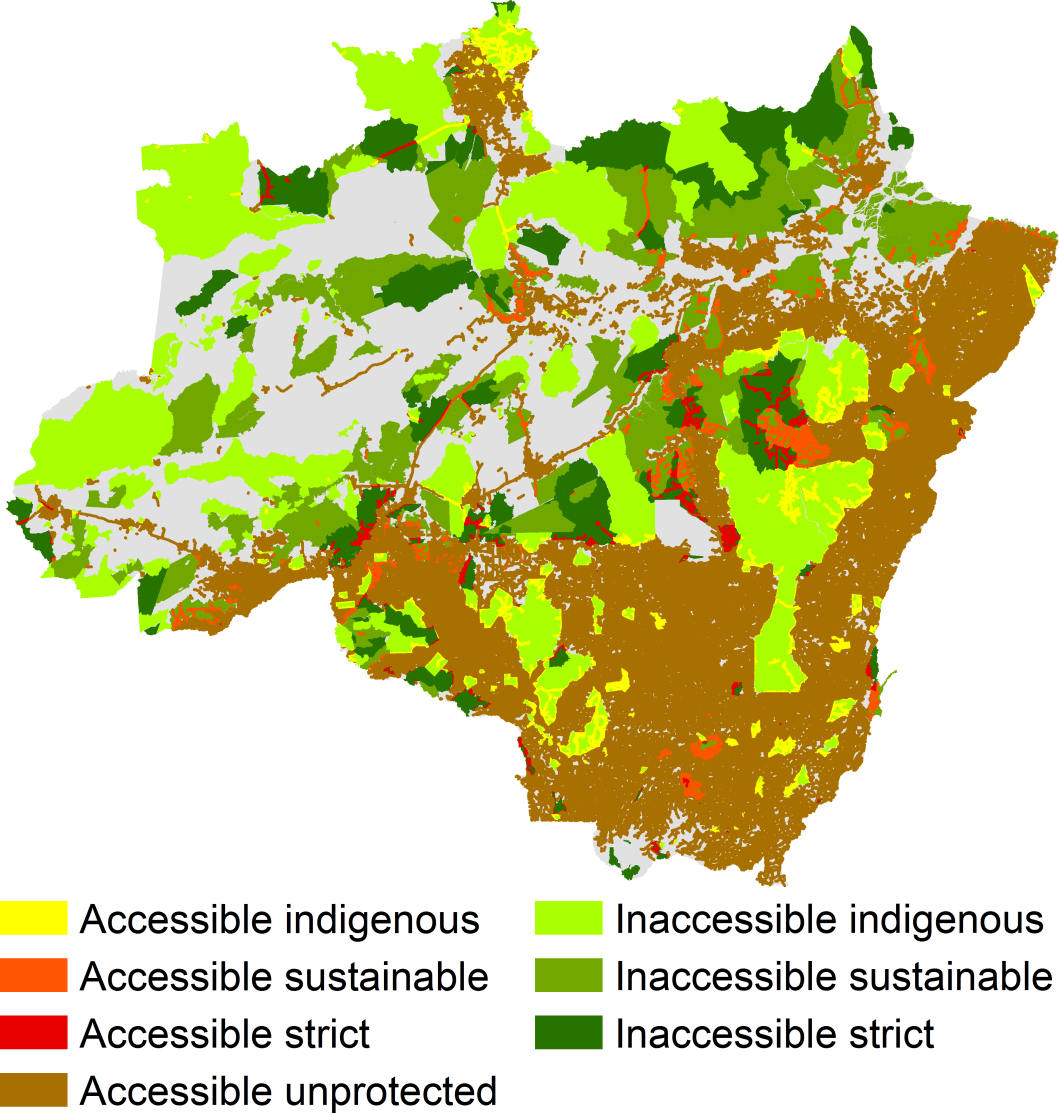
Accessible and inaccessible areas in seven states of Legal Amazon in 2010, by type of legal protection. Areas accessible by rivers are not shown in this map. Projection: Albers equal-area conic.

The accessible zone contained 98% of deforestation attributable to roads and provided access to 11.8% of protected surfaces in 2010; also, 10.1% of strictly protected areas, 13.1% of sustainable use areas, and 11.6% of indigenous lands were accessible. Illegal roads expanding to the west provide access to large areas in a larger complex of conservation units in central Pará (Fig 2). Also, relatively large territories of conservation units to the west of highway BR-163 in Pará and in northern Roraima are accessible, while many parks in the state of Amazonas are completely isolated.

### Avoided deforestation

Avoided deforestation estimates obtained by matching are more conservative than those obtained using the naïve approach, which subtracts the deforestation rate in unprotected areas from that of protected areas (Table 2). It is also evident that characteristics that explain deforestation (i.e., forest coverage, the road network) became increasingly dissimilar between unprotected and protected lands over the years (this is predominantly due to the fact that the treatment and the control samples change over the years as some previously unprotected lands become protected): during the 2001–2004 period the naïve approach overestimated avoided deforestation by 16% compared to the matching approach, by 42% during the 2005–2008 period, and by 92% during the 2009–2014 period. Interestingly, the naïve estimates suggest that the percentages of avoided deforestation were similar in strictly protected and indigenous lands during the 2005–2008 period, while matching indicates that indigenous lands avoided significantly more deforestation in percentage terms than strictly protected reserves during the same time.

**Table 2:** Average treatment effect on the treated (ATT) for accessible protected regions overall, and for accessible strict protection, sustainable use, and indigenous governance areas.

	ATT (%)	ATT (km^2^)	# matched pairs	MASMD (%)	ATT naïve (%)
*2001–2004 (≤ 2000)*					
All	-6.14	-5787	93616	2.86	-7.12
Strict	-4.44	-430	8735	7.41	-6.78
Sustainable	-3.71	-1073	28858	2.94	-5.68
Indigenous	-7.63	-4246	55650	2.17	-7.93
*2005–2008 (≤ 2000)*					
All	-2.6	-2448	94083	4.13	-3.72
Strict	-2.69	-266	8877	2.96	-3.94
Sustainable	-1.55	-453	29180	1.56	-2.76
Indigenous	-3.24	-1782	55036	8.57	-4.19
*2005–2008 (≤ 2004)*					
All	-2.56	-2707	105864	3.57	-3.63
Strict	-2.68	-307	10650	3.07	-4.02
Sustainable	-1.58	-557	35152	1.22	-2.56
Indigenous	-3.19	-1888	59263	7.79	-4.2
*2009–2014 (≤ 2000)*					
All	-1.11	-1072	96773	4.82	-2.09
Strict	-1.76	-176	8222	3.8	-2.69
Sustainable	-0.79	-241	30386	1.82	-1.13
Indigenous	-1.28	-724	56386	5.56	-2.5
*2009–2014 (≤ 2004)*					
All	-1.2	-1305	108820	4.49	-2.07
Strict	-1.95	-230	10148	3.97	-2.73
Sustainable	-1.02	-368	35838	2.38	-1.14
Indigenous	-1.31	-798	60869	5.18	-2.5
*2009–2014 (≤ 2008)*					
All	-0.91	-1606	177260	8.67	-1.75
Strict	-1.6	-603	35411	11.67	-2.55
Sustainable	-0.23‡	-153‡	64931	6.54	-0.6
Indigenous	-1.27	-930	73293	5.06	-2.39

The ‡ symbol implies that an estimate is not statistically significant at the 10% level (p value > 0.1), no symbol implies that an estimate is statistically significant at the 1% level (p value ≤ 0.01).

The ATT in square kilometers is estimated as ATT in percent, multiplied by the number of treatment observations in the sample. Mean absolute standardized mean difference (MASMD) is calculated as a simple average over absolute SMD values. A naïve estimate of the ATT is the difference between deforestation rates in accessible protected and accessible unprotected regions. Intervals (e.g., 2001–2004) imply a period to which the results correspond. Figures in parentheses (e.g., ≤ 2000) imply that only protected areas established in or prior to the specified year were included into the analysis.

During the 2001–2004 and 2005–2008 periods the most deforestation was avoided on indigenous lands, both in terms of percentage and area. This is consistent with earlier findings [10]. However, ATT percentages for the 2001–2004 period especially, and also for the 2005–2008 period, were slightly conservative because accessible zones were defined according to 2010 roads, meaning that some illegal roads were not present from 2001 to 2008. In other words, some *de facto* areas were classified as *de jure*: slightly reducing ATT percentages. The success of indigenous communities in saving forests is likely due to the active enforcement of legal restrictions on natural resource exploitation by outsiders and ongoing alliances between indigenous people and conservation organizations [35, 36].

Strictly protected areas consistently outperformed sustainable use areas in terms of deforestation avoidance rates. This finding contrasts with earlier studies [8, 9, 16] which found that protection type has little influence on avoided deforestation or fires. As already discussed, the reason for this is that strictly protected areas are more often located in low pressure areas compared to regions designated for sustainable use. The gap in deforestation avoidance rates between strictly protected areas and sustainable use areas has grown in recent years: during the 2001–2004 period, strictly protected areas only showed a 20% higher avoidance rate, while that rate was roughly 2.2 times greater during the last period (2009–2014) in those conservation units created in or prior to 2000. However, sustainable use areas protected a larger area of forests from deforestation. For instance, during the 2001–2004 period more than 1000 km^2^ of forests were saved by the sustainable use designation, which was more than twice the area saved by strict protection during the same time (ATT estimates in square kilometers are somewhat conservative because the study does not include areas accessible by rivers).

**Fig 3:**
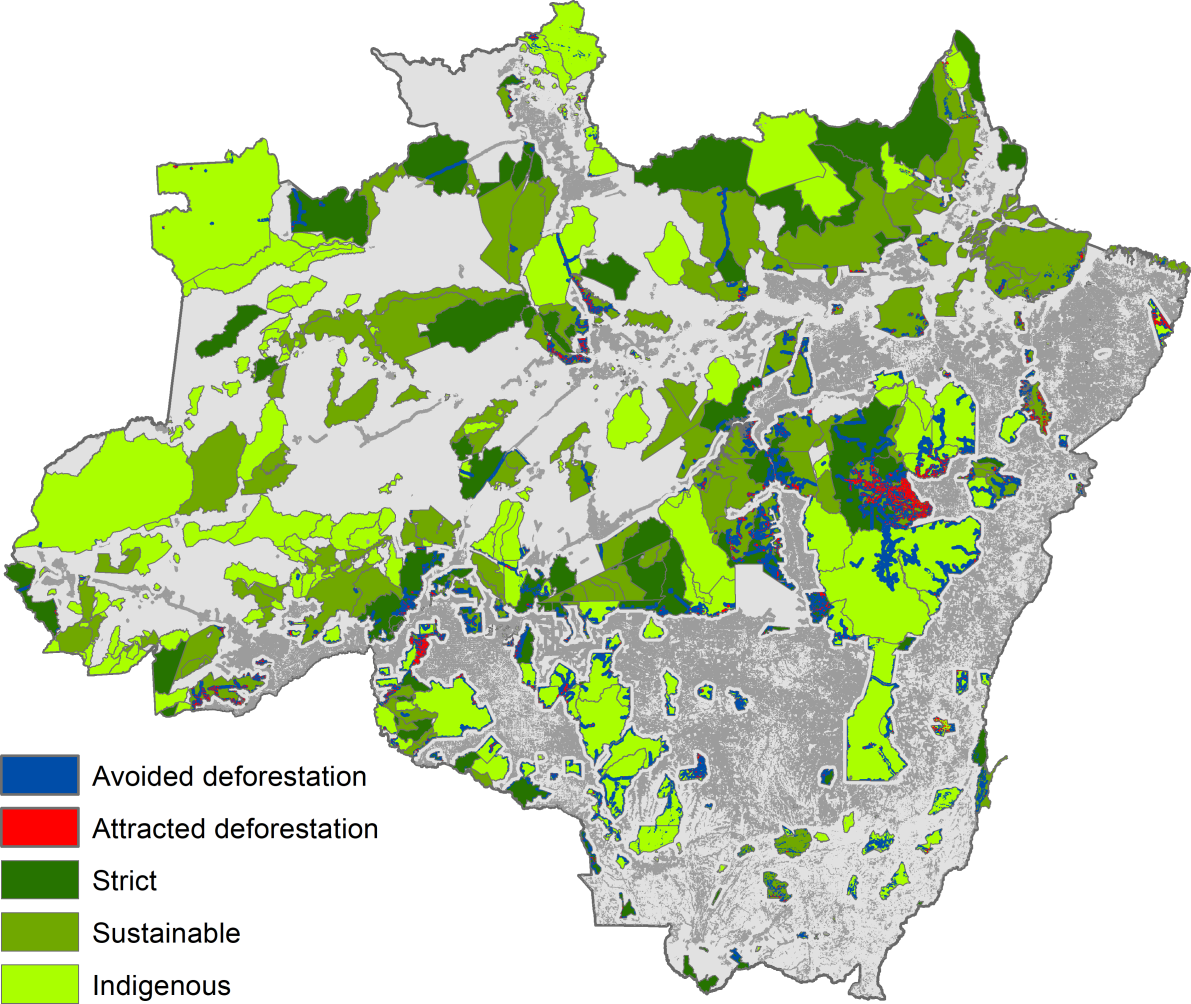
Regions that avoided and attracted deforestation during the 2009–2014 period. Only protected areas larger than 100 km^2^ in territory or created prior to or in 2008 are shown on maps. Military areas are not shown. Projection: Albers equal-area conic.

Several important changes emerged during the 2009–2014 period. Firstly, strictly protected areas outperformed indigenous lands in terms of the percent of saved forests. The second change is fundamental: Brazil’s network of sustainable use areas no longer avoided deforestation, on average. Nevertheless, sustainable use areas still avoided deforestation in parks created in or prior to 2004, indicating that location matters despite the fact that only accessible regions were studied. Also, during the 2009–2014 period, a couple of protected regions have emerged on the map where deforestation is attracted rather than avoided (clusters of land parcels that attract deforestation became clearly discernible on the map), specifically, in a large territory in the eastern Legal Amazon and a smaller area in the south-western part (Fig 3). The findings do not explain the process, it can only be speculated that protests against protection and immigration are likely causes of this result. Paradoxically, increasingly frequent [37] protected area downgrading, downsizing, and degazettement practices may benefit ecosystems in these regions. Location influence on deforestation avoidance rates validates the following paragraph, which analyzes the 2009–2014 period by splitting the data into subsamples based on the median values of each covariate.

Even though sustainable use areas did not contribute to deforestation avoidance on average during the 2009–2014 period, the findings revealed that regions at higher altitudes, in closer proximity to official roads and rivers, with less standing forests, or with lower precipitation levels avoided more than 1% of deforestation under the sustainable use designation during the same time in protected areas established in or prior to 2000 (Table 3). In regions with less standing forests, lower precipitation, or located near rivers sustainable use areas avoided more deforestation in percentage terms than indigenous lands, on average (1.28%, see Table 2). Strictly protected areas were especially successful in regions near logging roads where avoided deforestation exceeded the overall avoidance rate in strict protection areas during the 2009–2014 period by more than 70% (strictly protected areas established in or prior to 2000 avoided 1.76% of deforestation during the 2009–2014 period, on average, while regions from these areas that are in closer proximity to logging roads avoided 3.05% of deforestation during the same time). The findings also corroborate the common understanding that protected areas near roads avoid more deforestation than those further from roads [17, 18].

**Table 3:** Average treatment effect on the treated (ATT) for accessible protected areas for the 2009–2014 period by characteristics.

		elev	slope	forest	edge	prec	soil	rof	runf	river	time
All	Low	-1.29	-0.84	-1.39	-1.12	-1.25	-1.03	-1.11	-1.28	-1.52	-1.12
	High	-1.89	-1.54	-0.98	-0.75	-1.22	-1.15	-1.08	-0.93	-1.16	-1.15
Strict	Low	-2.21	-1.59	-1.61*	-1.76	-1.3	-1.59	-1.83	-3.05K	-1.36	-1.57
	High	-1.11K	-1.99	-1.68	-0.87*	-2.02	-1.85	-1.61	-1.25	-1.68	-2.04
Sustainable	Low	-0.82	-0.51‡	-1.34	-0.67K	-1.34	-0.89K	-1.07	-0.81‡	-1.44	-0.88
	High	-1.2	-0.96	-0.52‡	-0.69K	-0.33‡	-0.63K	-0.4‡	-0.78	0.43‡	-0.63‡
Indigenous	Low	-1.72	-1.03	-1.03	-1.39	-1.19	-1.19	-0.94	-1.8	-1.47	-1.29
	High	-2.03	-1.92	-1.29	-0.68	-1.71	-1.33	-1.34	-0.98	-1.83	-1.37

All protected areas created in or prior to 2000 were included. *Low* (or *High*) indicates that a subsample includes all parcels for which the values of a characteristic is below (or above) the median value of that characteristic. Unless accompanied by some symbol, ATT estimates are statistically significant at the 1% level (p value ≤ 0.01).

The *, K, and ‡ symbols imply statistical significance at the 5% level (0.01 < p value ≤ 0.05), at the 10% level (0.05 < p value ≤ 0.1), and statistical insignificance at the 10% level (p value > 0.1), respectively.

Covariate abbreviations appear in the same order as in Table 1 (refer to Table 1 to link abbreviations with covariate names and descriptions). Refer to Table B in S2 File for detailed results.

### Standardized mean differences

**Fig 4:**
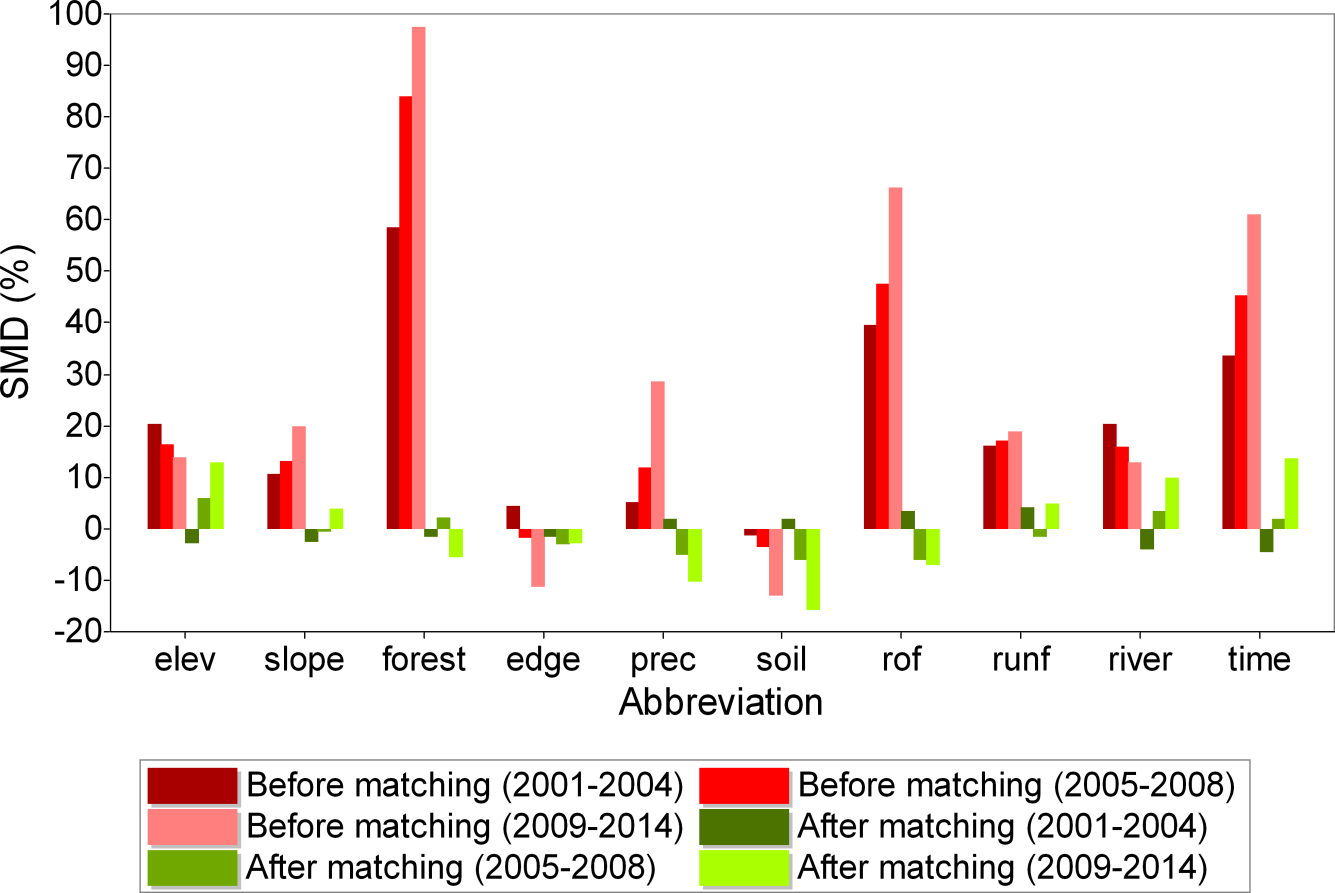
Standardized mean differences (SMDs) before and after matching for different covariates and periods. A sample corresponding to a period includes protected areas created prior to that period. Covariate abbreviations on the x-axis are listed in the same order as in Table 1 (refer to Table 1 to link abbreviations with covariate names and descriptions).

Red columns in Fig 4 reflect the level of non-randomness in the location of protected areas. It is evident that the matching process found control cells with characteristics similar to those of the treatment cells (see Figure C in S2 File for geographical distribution of matched control cells). Matching was especially successful for the 2001–2004 period due to a larger control sample. As for the 2009–2014 period, treatment and matched control samples had noteworthy, though still minor, differences in elevation, agricultural suitability, and travel time. Despite these differences, overall bias (simple average over absolute SMD values) was below 10% for all protection types and periods, except on one occasion (Table 2). For some subsamples based on covariate values (low vs. high), accurate pairs from the control group could not be found. But, there were relatively few of these (Table B in S2 File).

## Discussion

The results may provide valuable information for policy planning if interpreted correctly. It is important to understand that the results do not necessarily reflect the relative effectiveness of each protection type, because the characteristics that explain deforestation may be different on average in strictly protected, sustainable use, and indigenous governance areas. Also, it is a cross-sectional analysis over different time periods, but the results are not comparable between the periods, i.e., lower deforestation avoidance rates in the subsequent periods do not imply that legal protection became less effective, because pre-trends in processes that explain deforestation are unlikely parallel. Deforestation avoidance in the early 2000s was substantial. Later, effective policies for curbing deforestation were put into place along with networks of protected areas, most notably the Soy Moratorium [38], the Beef Moratorium [39], and rural credit constraints [40]. These policies aggravated agricultural expansion, but to a different extent on protected and unprotected lands. For instance, as strictly protected areas prohibit agriculture, new policies had a marginal effect on curbing deforestation in these areas, but the effect was substantial on unprotected lands, where agriculture is the dominant land use.

The finding that sustainable use areas no longer avoid deforestation on average will likely invigorate debates over a novel sustainable development paradigm [41] and country-wide payment scheme [42]. However, this study showed that, in regions with certain characteristics, sustainable use areas can avoid more deforestation than indigenous lands, on average. Thus, selecting the proper location for these areas is very important. For example, sustainable use areas created in less densely forested regions continue to contribute to deforestation avoidance. Even sustainable use areas that do not avoid deforestation should not be devalued, because a comprehensive analysis of park benefits must consider other potential benefits of legal protection, such as avoiding selective logging, poaching, gold mining, and illegal road proliferation, as well as their problems, such as ecosystem isolation and edge effects [43, 44]. Even if legal protection does not avoid deforestation in an accessible area, it may prevent illegal roads from penetrating forests, thereby preventing farmer colonization and subsequent deforestation into areas that are inaccessible thanks to the protection status. This *shadow* avoided deforestation is difficult to quantify, but its contribution to deforestation avoidance might be substantial.

This study unveiled important changes in deforestation avoidance patterns: 1) strictly protected areas have overtaken indigenous lands in terms of deforestation avoidance rate, 2) some protected regions started to attract deforestation instead of avoiding it, and most importantly, 3) sustainable use areas no longer avoid deforestation on average. The findings also raised new questions. Why do sustainable use areas no longer generally inhibit deforestation? Have strictly protected areas started to avoid more deforestation than indigenous lands because of newly adopted policies that affect deforestation patterns to a different extent under the two types of legal protection, or have indigenous people adopted the values of modern civilization, which often prioritizes economic wealth over nature conservation? Understanding the processes behind new patterns is a direction for future research.

## Supporting information

S1 File (7Z)

S2 File (PDF)
