# Supplementary material for: Changing patterns in deforestation avoidance by different protection types in the Brazilian Amazon
Source: PLoS One. 2018 Apr 24;13(4):e0195900. doi: 10.1371/journal.pone.0195900 (PMC5918171; doi:10.1371/journal.pone.0195900)
Supplement: S2 File — (PDF) [file pone.0195900.s002.pdf]

Supporting information

GIS data processing

Deforestation. A land use raster provided by the National Institute of Space Research, INPE [1], under the PRODES project and with data from 2014 was downloaded and reclassified into deforestation, coded as 1, and other land uses, coded as 0. Larger parcels containing 16 times 16 PRODES parcels were formed. The values within each larger parcel were averaged and then resampled to a 1-km spatial resolution by bilinear interpolation.
Elevation and slope. The data, sourced from the Shuttle Topography Radar Mission, SRTM [2], is available from ~90 m^2 spatial resolution, once projected. Larger parcels containing 11 times 11 SRTM parcels were formed. The values within each larger parcel were averaged and then resampled by bilinear interpolation. Slope was estimated at the original spatial resolution using the *slope* tool in ArcGIS. The data was resampled to a 1-km spatial resolution by bilinear interpolation.
Forest cover. The data is distributed by NASA [3] as product MOD44B, version 051, and was obtained using *reverb*, a metadata and service discovery tool. The rasters contain yearly forest cover estimates as percentages at a ~250-meter spatial resolution. The 2001, 2005, and 2009 products (corresponding to the beginning of each study period) were downloaded. Water was assigned a value of 0. Larger parcels containing 4 times 4 MOD44B parcels were formed. The values within each larger parcel were averaged and then resampled by bilinear interpolation.
Forest edge. Deforestation that took place during or after a study period was reclassified as forests in the PRODES raster and added to the existing forest cover class. The Euclidean distance from each cell to the nearest forested cell was estimated at the original ~60-meter spatial resolution. Next, the data was processed in the same way as deforestation data.
Precipitation. The raster data is distributed by World-Clim [4] and is available as a multiyear average with monthly coverage at a ~1-km spatial resolution once projected. Yearly estimates were obtained by aggregating values of monthly precipitation. The data was resampled by bilinear interpolation. This data reflects general precipitation trends, and was treated as static. Alternative data, collected under NASA's Tropical Rainfall Measuring Mission (TRMM) provided yearly precipitation data but at a coarse spatial resolution (~25 km once projected). Therefore, the former was preferred. Since all study periods cover at least 4 years, actual precipitation over these periods should not have differed substantially from the general trends.
Agricultural suitability. The product **'**Climate, soil and terrain slope constraints combined' (Plate 28, Global agro-ecological zones), distributed by the International Institute for Applied System Analysis, IIASA [5], has 7 levels, where a 1 indicates a very high suitability for agriculture and a 7 indicates unsuitable lands. The data is available from a ~10-km spatial resolution once projected. It was resampled to a 1-km spatial resolution via the nearest neighbor method.
Official and unofficial roads. The shapefile of roads, compiled by the Amazon's Institute of Man and Environment, was partitioned into a shapefile of official roads and a shapefile of logging roads which included both unofficial and settlement roads. Distances to the nearest road were estimated as Euclidean distances from the center of each 1-km^2 cell to the nearest polyline representing a road. Since the road network also exists beyond the spatial extent of IMAZON's shapefile, some estimates along the borders of the study area are inaccurate. However, this imperfection should not have introduced substantial changes in the results. Another shortcoming

is related to temporal extent. Accessible regions defined by the study correspond to the year 2010. IMAZON provides information on which year each road segment was mapped. This information could be treated as a year when a logging road was built. A valid approach would be to select a segment of roads at the beginning of each study period, but this could not be done for the first period because the mapping of illegal roads began in 2003. In any case, this approach would not include deforestation that took place near logging roads built *during* a study period. For these reasons, accessible regions were defined only once and applied to all periods (but I acknowledge that Legal Amazon is dealing with the rapid proliferation of illegal roads [6]), implying that avoided deforestation estimates for the first two periods are conservative.

Rivers. A set of polylines representing navigable rives in Brazil was obtained from Brazil's National Map of Logistics and Transport, PNLT [7]. Distances were calculated in the same way as roads. The entire river shapefile was used in computations so as to minimize inaccuracies in estimates for cells located near the edges of the study area.

Travel time. Global Environment Monitoring Unit [8] provides a ~1 km spatial resolution raster with travel time estimates. For this study, they were resampled by bilinear interpolation.

Protected areas. The shapefiles of protected areas, distributed by Brazil's Ministry of Environment, MMA [9], were used to select all protected areas that fully or partially fell within the boundaries of the study area. The World's Database of Protected Areas, WDPA [10], was used to remove protected areas for which the actual reported physical area was zero and to assign IUCN (International Union for Conservation of Nature) categories. It must be noted that for some records in the attribute table of the WDPA shapefile, IUCN categories were not reported or were reported incorrectly. This data was cross-checked and, if necessary, corrected using Brazil's National Cadaster of Conservation Units, made available by the MMA. IUCN categories were also collected for those conservation units that were included by the MMA, but not included in the WDPA. The overlapping of polygons was handled according to WDPA recommendations. That is, strict protection areas were prioritized over other protection types and sustainable use areas were prioritized over indigenous lands. In case protected areas of the same protection type were overlapping, priority was given to the one created less recently. Information regarding the year a protection status was issued came from the MMA, as the WDPA reports the year when the current status (proposed, designated, or established) of the protected area came into force. If this data from the WDPA were used, some protected territories would be assigned to the control sample, thereby deflating avoided deforestation estimates.

Pixel selection

Accessibility. Accessibility was defined following Barber et al. [11]. Firstly, all deforestation pixels corresponding to the 2000-2014 period were allocated either to roads (not differentiating between official and unofficial ones) or to rivers using the shortest Euclidean distance as the allocation criterion. In this way, 95% of deforestation was attributed to roads, and the remainder was assigned to rivers. The shortest Euclidean distances from each deforestation pixel attributed to roads were calculated and placed into 100-meter intervals, and the accumulated percentage of deforestation in total deforestation was estimated for each interval (see Fig A for graphical illustration). The data was first differenced by subtracting the accumulated percentage of deforestation at distance $d+0.5$ km from the corresponding figure at $d-0.5$ km and centering the estimates at distance d. Since the range of each distance interval is 1 km, the result of the first

difference is also the slope at distance *d*. The distance at which this slope equals the slope of the diagonal line defines the distances at which deforestation penetration starts to diminish. The slope of the diagonal line is $100/d_{max}$, which is the slope of the red line in Fig A as well. Regions beyond the distance at which deforestation influence starts to diminish (4.1 km) were considered inaccessible.

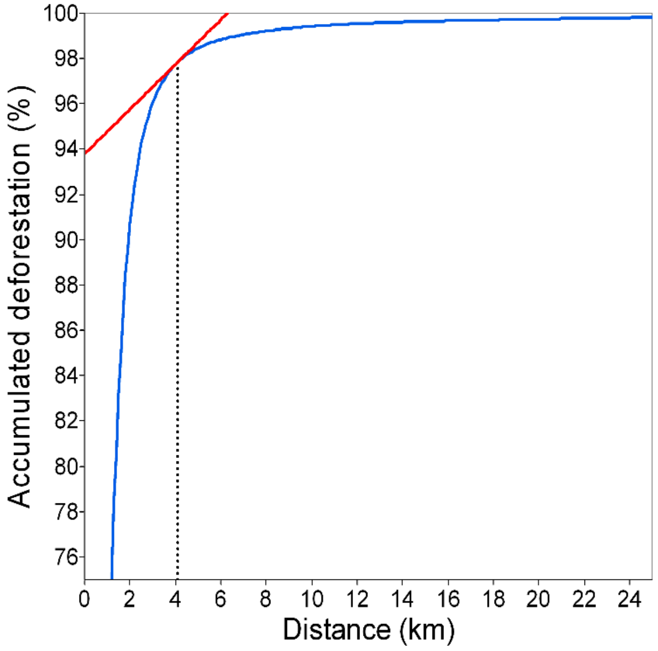

Fig A. Accumulated deforestation at various distances from roads in seven states of Legal Amazon. The axes are shortened to improve visualization. The red line is parallel to the main diagonal. The point, where the accumulated deforestation curve and the red line touch is the distance at which deforestation penetration starts to diminish (4.1 km).

Forest cover. Sparsely forested (<20%) cells were defined using a processed raster of forest cover and were removed, thus also removing parcels of agricultural lands, depleted areas, urban centers, and hydrographic features.

Clouds. To minimize cloud cover in the 2014 PRODES raster, pixels classified as forests in the 2015 PRODES raster, but classified as clouds in the 2014 version, were reclassified as forests in the 2014 PRODES raster. Next, the remaining clouded pixels were coded as 1 and other classes were assigned a value of 0. The final cloud layer was obtained by averaging these values and resampling the result into 1-km^2 land parcels. All parcels with at least 20% cloud cover were considered inadequate for matching and, therefore, were discarded.

Leakage. Leakage is defined as the displacement of extractive efforts from within protected areas into the broader landscape [12]. This definition can be widened to include deforestation from immigration to protected area surroundings. Even though empirical analyses suggest no leakage in Brazil [13], cells located within 10 kilometers of a protected area larger than 100 km^2 were discarded as a precaution. Retaining land parcels located close to protected areas would inflate avoided deforestation estimates if treatment cells were paired with cells affected by leakage. However, discarding these parcels reduced the size of control sample, thereby reducing the probability that proper matches could be found.

Borders. The cells were allocated to treatment and control samples based on cell center location. It is possible that a cell was predominantly located just outside the borders of a protected area and was affected by rampant deforestation due to leakage, while its center lay within the borders of that protected area. To hedge against such a possibility, cells that cover both protected and unprotected lands were removed.

Only cells that satisfied all five conditions were considered eligible for matching (Fig B). Cells that were located within protected areas for which less than 100 suitable observations were found in the sample of eligible observations or in those protected areas created during a study period, were removed programmatically. However, cells of those protected areas which were created after a study period were kept in the control group of that period. Descriptive statistics for parcels eligible for matching are presented in Table A.

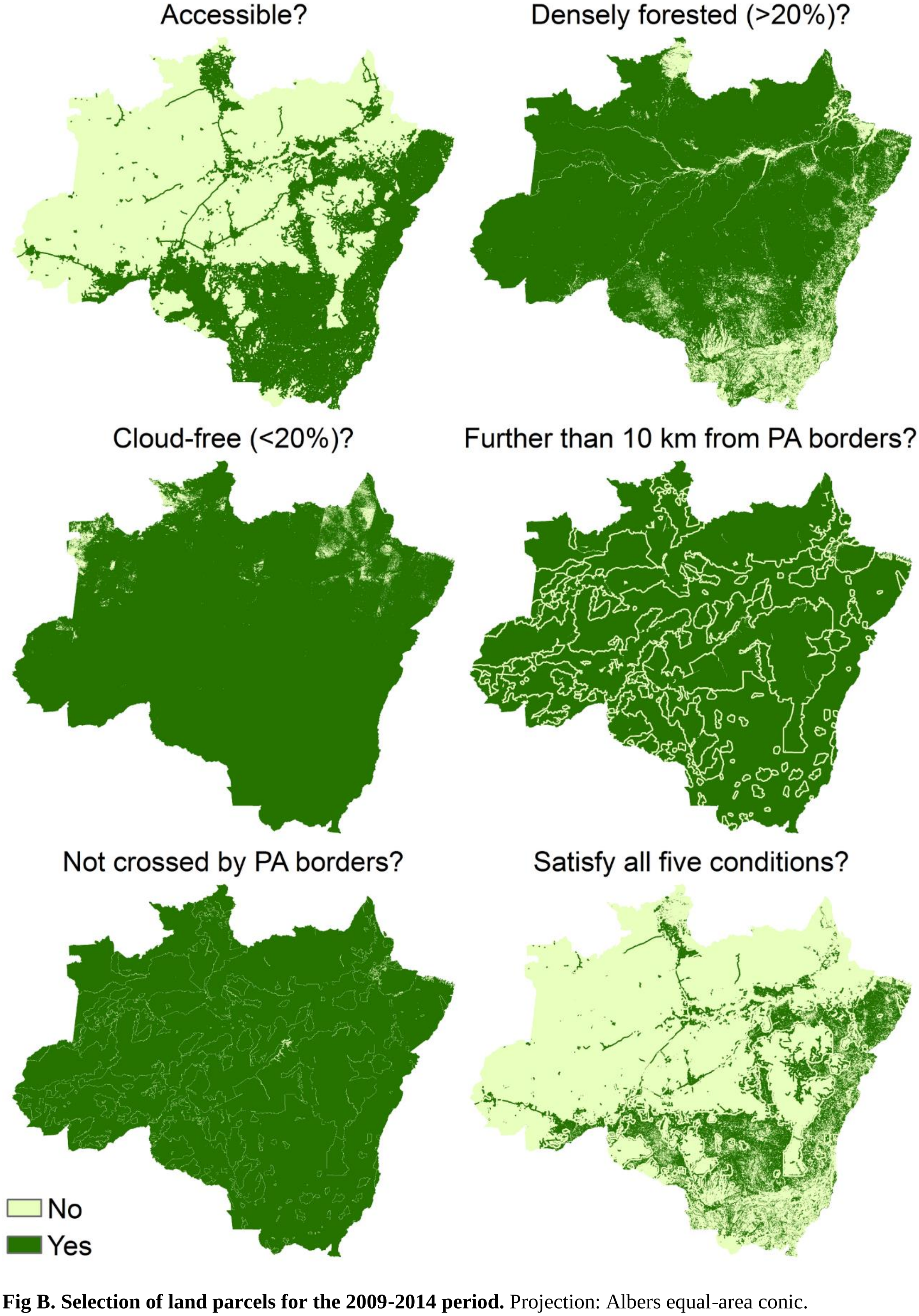

Fig B. Selection of land parcels for the 2009-2014 period. Projection: Albers equal-area conic.

Table A. Descriptive statistics for land parcels in treatment, control, and matched control groups. Refer to Table 1 for units of measurement. For static variables, the statistics correspond to the 2009-2014 period (minor differences between the periods exist because the number of protected areas is different for the three periods).

	Mean	Standard deviation	Minimum	Maximum
**Treatment group**				
deforestation 2001-2004	1.57	7.13	0	100
deforestation 2005-2008	0.98	5	0	99.57
deforestation 2009-2014	1.22	5.51	0	100
elevation	240.11	127.37	3.28	1009.83
slope	3.24	2.72	0	31.36
forest cover 2001-2004	65.69	15.85	20.01	83.84
forest cover 2005-2008	67.58	16.57	20	84.69
forest cover 2009-2014	65.56	16.67	20	86.35
forest edge 2001-2004	899.73	4460.08	0	50334.6
forest edge 2005-2008	707.35	3602.71	0	50334.6
forest edge 2009-2014	668.18	3420.28	0	50334.6
precipitation	2069.74	316.26	0	3672
agricultural suitability	5.82	1.07	1	7
official roads	57976.52	53408.35	0	262458
unofficial roads	4584.77	17567.94	0	238321
rivers	48076.11	42375.66	0	269765
travel time	1411.69	1336.42	0	8580
**Control group**				
deforestation 2001-2004	8.7	17.58	0	100
deforestation 2005-2008	4.62	11.42	0	100
deforestation 2009-2014	2.98	7.8	0	100
elevation	222.27	132.06	0	1060.81
slope	2.7	2.09	0	26.49
forest cover 2001-2004	56.39	18.69	20	84.46
forest cover 2005-2008	53.63	19.96	20	84.68
forest cover 2009-2014	49.32	19.74	20	86.52
forest edge 2001-2004	697.48	3211.82	0	67949.1
forest edge 2005-2008	768.92	3233.67	0	67949.1
forest edge 2009-2014	1053.07	3941.71	0	70408.1
precipitation	1979.15	278.05	0	3744
agricultural suitability	5.96	0.96	1	7
official roads	22578.54	31946.25	0	433721
unofficial roads	1256.73	1671.25	0	70214
rivers	42625.6	38644.27	0	230835
travel time	594.17	541.21	0	6038

Table A. Cont.

**Matched control group**				
deforestation 2001-2004	7.72	17.57	0	100
deforestation 2005-2008	3.54	10.63	0	100
deforestation 2009-2014	2.13	7.32	0	100
elevation	223.54	141.27	0.27	1060.81
slope	3.13	2.89	0.03	26.49
forest cover 2001-2004	65.84	14.65	20	84.44
forest cover 2005-2008	67.23	15.77	20	84.68
forest cover 2009-2014	66.48	15.97	20	86.52
forest edge 2001-2004	977.55	4784.82	0	63740.8
forest edge 2005-2008	818.64	4353.57	0	67949.1
forest edge 2009-2014	767.38	4407.5	0	70408.1
precipitation	2102.3	287.63	0	3732
agricultural suitability	5.99	0.93	1	7
official roads	61712.3	83397.85	0	433721
unofficial roads	3700.06	8017.68	0	70214
rivers	43891.68	38943.22	0	230413
travel time	1230.41	961.18	0	6038

Results

Table B. Total number of matched pairs (NM) and mean absolute standardized mean difference (MASMD) for accessible protected areas for the 2009-2014 period by characteristics. All protected areas created in or prior to 2000 were included. *Low* (*High*) indicates that a subsample includes all parcels for which the values of a characteristic is below (above) the median value of that characteristic.

NM		**elev**	**slope**	**forest**	**edge**	**prec**	**soil**	**rof**	**runf**	**river**	**time**
All	Low	41505	45929	17979	75311	44633	29518	31267	14052	44685	33107
	High	55237	50844	78713	21403	49682	37920	65250	64115	51847	63611
Strict	Low	4825	4198	1082	6258	4620	2589	3504	732	4649	1680
	High	2704	3530	6983	1583	2737	2820	4158	6312	2866	6748
Sustainable	Low	19651	11765	6126	24130	12440	7169	10742	5764	16759	15377
	High	10474	18607	24140	6229	17478	15546	19379	17578	13492	14931
Indigenous	Low	14452	28109	10450	42970	26799	19015	14672	7270	22586	15413
	High	41480	28247	45796	13320	27571	19304	41492	38543	33432	40882
MASMD											
All	Low	4.62	5.51	4.2	7.02	7.28	11.96	5.59	6.59	4.31	3.63
	High	12.58	5.99	6.31	3.98	8.13	5.71	6.47	5.24	8.07	7.41
Strict	Low	3.57	3.12	11.08	3.15	6.07	5.5	4.93	5.48	4.16	7.75
	High	5.79	6.75	4.54	9.41	7.01	4.31	2.69	5.77	7.01	7.21
Sustainable	Low	4.03	7.35	8.41	1.76	2.53	4.03	4.18	2.74	2.11	2.72
	High	4.21	1.82	2.1	12.68	2.69	2.88	1.89	1.93	3.85	4.48
Indigenous	Low	6.39	5.22	3.26	10.06	10.82	21.53	4.79	11.8	6.98	3.93
	High	27.28	11.23	8.02	4.31	19.27	2.55	6.67	4.65	25.24	9.2

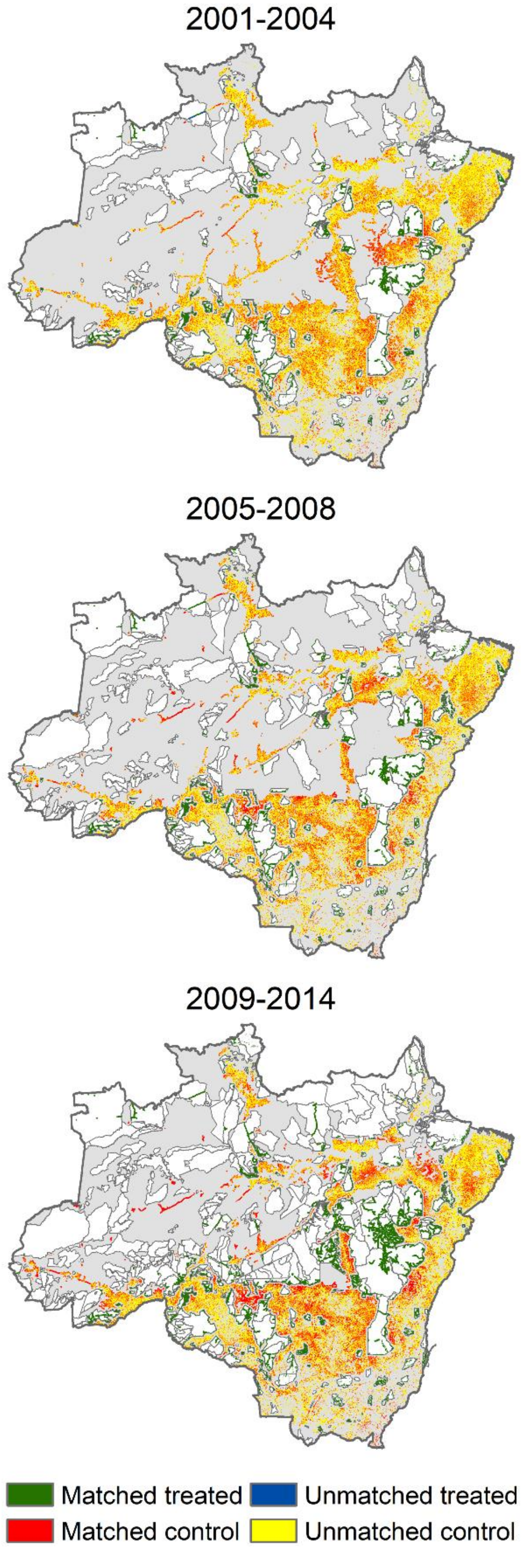

Fig C. Spatial distribution of matched treated, unmatched treated (observations outside caliper), matched control, and unmatched control cells. Projection: Albers equal-area conic.

References

1. INPE. Projeto PRODES, Monitoramento da Floresta Amazônica Brasileira por Satélite (Instituto Nacional de Pesquisas Espaciais, São José dos Campos, Brazil). 2016. Available from www.obt.inpe.br/prodes/index.php.
2. SRTM-NASA. 90m Digital Elevation Data, Version 4 (Shuttle Radar Topography Mission-National Aeronautics and Space Administration, Washington, DC, USA). 2016. Available from http://srtm.csi.cgiar.org/.
3. NASA. MODIS Vegetation Continuous Fields Yearly L3 Global 250m, Version 051 (National Aeronautics and Space Administration, Washington, DC, USA). 2016. Available from https://reverb.echo.nasa.gov/reverb/.
4. Hijmans RJ, Cameron SE, Parra JL, Jarvis A. Very high resolution interpolated climate surfaces for global land areas International Journal of Climatology. 2005;25(15):1965–1978. Available from www.worldclim.org.
5. IIASA. Plate 28-Climate, soil and terrain slope constraints combined (International Institute for Applied System Analysis, Laxenburg, Austria). 2016. Available from http://webarchive.iiasa.ac.at/Research/LUC/GAEZ/.
6. Ahmed SE, Souza CM, Riberio J, Ewers RM. Temporal patterns of road network development in the Brazilian Amazon. Regional Environmental Change. 2013;13(5):927–937.
7. PNLT-MT. Hidrovias (Plano Nacional de Logística e Transportes-Ministério dos Transportes, Brasília, Brazil). 2016. Available from http://pnlt-imagem-govfed.opendata.arcgis.com/.
8. Nelson A. Travel Time to Major Cities: A Global Map of Accessibility (Global Environment Monitoring Unit, Joint Research Centre of the European Commission, Ispra, Italy). 2008. Available from http://forobs.jrc.ec.europa.eu/products/gam/.
9. MMA. Download de dados geográficos (Ministério do Meio Ambiente, Brasília, Brazil). 2017. Available from http://mapas.mma.gov.br/i3geo/datadownload.htm.
10. UNEP-WCMC. World Database of Protected Areas (United Nations Environmental Programme-World Conservation Monitoring Centre, Cambridge, UK). 2016. Available from www.protectedplanet.net.
11. Barber CP, Cochrane MA, Souza CM, Laurance WF. Roads, deforestation, and the mitigating effect of protected areas in the Amazon. Biological Conservation. 2014;177:203–209.
12. Bode M, Tulloch AIT, Mills M, Venter O, Ando AW. A conservation planning approach to mitigate the impacts of leakage from protected area networks. Conservation Biology. 2015;29(3):765–774.
13. Soares-Filho BS, Moutinho P, Nepstad D, Anderson A, Rodrigues H, Garcia R, et al. Role of Brazilian Amazon protected areas in climate change mitigation. Proceedings of the National Academy of Sciences. 2010;107(24):10821–10826.
